# Risk factors for unstable blood glucose level: integrative review of the risk factors related to the nursing diagnosis

**DOI:** 10.1590/1518-8345.1688.2893

**Published:** 2017-06-05

**Authors:** Andressa Magalhães Teixeira, Rosangela Tsukamoto, Camila Takáo Lopes, Rita de Cassia Gengo e Silva

**Affiliations:** 1 RN.; 2 MSc, RN, Hospital Universitário, Universidade de São Paulo, São Paulo, SP, Brazil.; 3 PhD, Professor, Escola Paulista de Enfermagem, Universidade Federal de São Paulo, São Paulo, SP, Brazil.; 4 PhD, Professor, Escola de Enfermagem, Universidade de São Paulo, São Paulo, SP, Brazil.

**Keywords:** Diabetes Mellitus, Type 2, Nursing Diagnosis, Risk Factors, Hypoglycemia, Hyperglycemia

## Abstract

**Objective:**

to identify evidence in the literature on the possible risk factors for the risk of unstable blood glucose diagnosis in individuals with type 2 diabetes mellitus, and to compare them with the risk factors described by NANDA International.

**Method:**

an integrative literature review guided by the question: what are the risk factors for unstable blood glucose level in people with type 2 diabetes mellitus? Primary studies were included whose outcomes were variations in glycemic levels, published in English, Portuguese or Spanish, in PubMed or CINAHL between 2010 and 2015.

**Results:**

altered levels of glycated hemoglobin, body mass index>31 kg/m^2^, previous history of hypoglycemia, cognitive deficit/dementia, autonomic cardiovascular neuropathy, comorbidities and weight loss corresponded to risk factors described in NANDA International. Other risk factors identified were: advanced age, black skin color, longer length of diabetes diagnosis, daytime sleepiness, macroalbuminuria, genetic polymorphisms, insulin therapy, use of oral antidiabetics, and use of metoclopramide, inadequate physical activity and low fasting glycemia.

**Conclusions:**

risk factors for the diagnosis, risk for unstable blood glucose level, for persons with type 2 diabetes mellitus were identified, and 42% of them corresponded to those of NANDA International. These findings may contribute to the practice of clinical nurses in preventing the deleterious effects of glycemic variation.

## Introduction

*Risk for unstable blood glucose level* (00179) is a NANDA International, Inc. (NANDA-I) nursing diagnosis (ND), defined as "Vulnerable to variation in blood glucose/sugar levels from the normal range, which may compromise health"^1^.

In the latest NANDA-I diagnostic classification edition, 16 risk factors for this ND are described: alteration in mental status, average daily physical activity is less than recommended for gender and age; compromised physical health status; delay in cognitive development; does not accept diagnosis; excessive stress; excessive weight gain; excessive weight loss, inadequate blood glucose monitoring; ineffective medication management; insufficient diabetes management; insufficient dietary intake; insufficient knowledge of disease management; nonadherence to diabetes management plan; pregnancy; and rapid growth period^2^ ^-^ ^3^ ^)^ , which are used to identify this diagnosis in patients of different clinical profiles or health-disease conditions. 

Among these conditions, a special interest in type 2 diabetes mellitus (DM2) is demonstrated in this study. In a study that investigated 30 people during home nursing consultations, 60% had unstable glycemic risk^4^. In another study with diabetic patients in outpatient care, 28.6% of the participants had this ND^5^.

Studies demonstrate that variation in glycemic levels may: increase the rate of complications and mortality in hospitalized patients with acute coronary syndrome^6^, compromise renal function and structure^7^, and lead to endothelial dysfunction^8^. These consequences can have negative impact on productivity, quality of life and survival, and involve high costs related to treatment^9^. Thus, the recognition of risk factors for unstable glycemia and the institution of preventive measures can contribute to positive results for which nurses have responsibility.

The recognition of the risk factors of this ND may occur due to the nurse's knowledge, his previous experiences, and by means of consultation of available scientific literature, among others. In clinical practice, NANDA-I diagnosis classification is an important, easily accessible resource that guides nurses in recognizing risk factors, and in the clinical decision-making process.

The movement of researchers to improve the NANDA-I diagnosis classification, including new diagnostic elements, is current in the literature. In a literature review, 79 defining characteristics of the diagnosis of *decreased cardiac output* (00029) were identified, of which 28 were approved by NANDA-I and the others were identified as possible indicators of this diagnosis^10^. In another study, researchers found that the distances in the six-minute walk test were predictive of *ineffective peripheral tissue perfusion* (00204) and suggested that this could be a defining characteristic of that diagnosis^11^.

*Risk for unstable blood glucose level* is supported by three references published between 2003 and 2005^1^. Therefore, reviewing the ND and supporting its elements, such as risk factors, in current literature is of fundamental importance.

In this context, the objectives of this study were to identify evidence in the literature regarding possible risk factors for the diagnosis of *risk for unstable blood glucose level* for individuals with DM2, and to compare them with the risk factors described by NANDA-I.

## Method

This literature integrative review was conducted according to the following steps: identification of the research question; definition of the criteria for inclusion and exclusion of studies; categorization and evaluation of studies; extraction and interpretation of results; and synthesis of knowledge ^12^. The question that led to the survey data was: What are the risk factors for unstable glycemia in people with type 2 diabetes mellitus?

**Figure 1:**
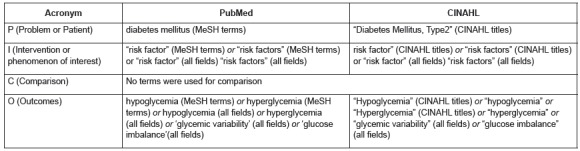
Search strategy used. São Paulo, SP, Brazil, 2015

The PICO [P(problem or patient) I (intervention) C (comparison) O (outcomes)] acronym^13^ was used to develop strategies for searching the PubMed portal (National Library of Medicine and the National Institutes of Health) and CINAHL database (Cumulative Index to nursing Allied Health Literature), as described in Figure 1. These two databases were chosen because they include the main journals in the health and nursing areas that involve the subject of interest for the present study. 

The search was performed from October to November of 2015. Initially, the terms "risk factor" and "risk factors" were used in the search strategy, but results obtained were nonspecific. The use of these terms provided articles in which unstable glycemia was a risk factor for other diseases. Therefore, these terms were suppressed, which made the search results more specific and better answered the research question and, therefore, its replacement was not necessary.

In order to be included in this review, the studies needed to meet the following inclusion criteria: to investigate variation in blood sugar levels that may compromise health as an outcome, defined as that which increases or decreases serum glucose; be a primary study with a longitudinal design (retrospective or prospective cohort, or case-control), cross-sectional study (in which the causal relationship between the antecedent variable and the outcome was clear), or an experimental study; have an abstract and full text available in the databases mentioned above; published between 2010 and 2015, in Portuguese, English or Spanish; and have adequate methodological quality.

An adequate methodological quality was assessed according to a consistency with 50% or more of the items in the STROBE (Strengthening the Reporting of Observational Studies in Epidemiology) Statement as Barbosa, Vasconcelos, Correia, & Ferreira^14^ and Silva, Lyra, & Lima^15^ used in their studies. 

This tool was used because it guides the organization of scientific writing of observational studies, indicating essential elements that must be contained in the manuscripts. Articles with an agreement of 50% or more of the STROBE items were considered to have adequate methodological quality. This evaluation was performed by two evaluators, independently, and inconsistencies were resolved by consensus.

The level of evidence from included studies was assessed according to the *Oxford Centre for Evidence-based Medicine* classification for etiology: 2b: cohort study, 3b: case-control study; 4: studies without clear definition of comparison groups that do not measure exposure and outcome, without patient follow-up (used to classify cross-sectional studies)^16^. 

**Figure 2:**
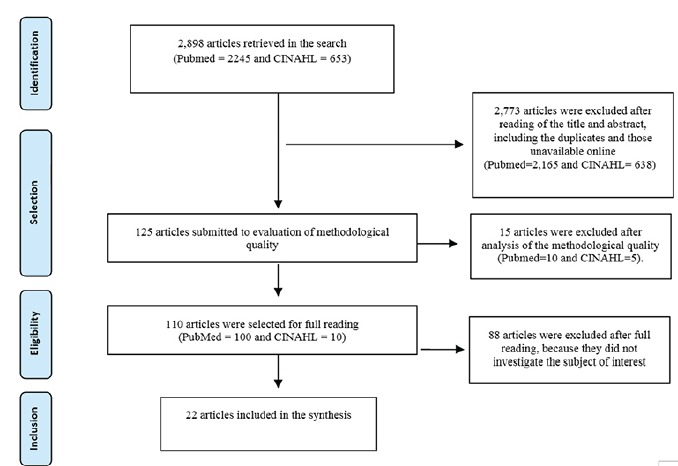
Flow chart of the study selection process.

The studies excluded were those testing the efficacy or effectiveness of medications, measures for glycemic control, and studies including people with other types of diabetes with results that did not evaluate those with T2DM separately. Figure 2 shows a summary of the study selection process.

After this classification, possible correspondences with the risk factors currently described by NANDA-I were evaluated by consensus among the researchers.

 For the extraction of the data of interest, an instrument developed by the researchers was used, containing: title; objective; design; sample; results; and risk factors identified in the article. Data were extracted by two evaluators independently. Inconsistencies were resolved by consensus between the two.

Risk factors were classified as factors associated with a greater likelihood of increasing blood glucose levels, and factors associated with a greater likelihood of lowering blood glucose levels.

## Results

**Figure 3:**
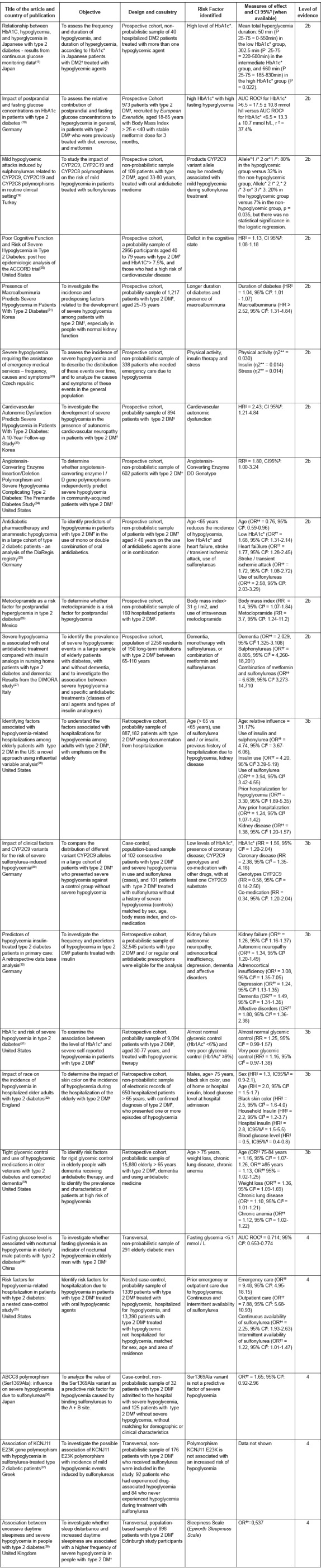
Characteristics of the selected articles. São Paulo, SP, Brazil, 2015.

Twenty-two primary studies met the eligibility criteria. All were published in English or Spanish. The countries of origin of the articles were the United States (n=6), Germany (n=4), Japan (n=2), Korea (n=2), United Kingdom (n=1), Turkey (n=1), Czech Republic (n=1), Greece (n=1), Mexico (n=1), Italy (n=1), China (n=1). and England (n=1). The characteristics of these studies are demonstrated in Figure 3. 

*HbA1c: Glycated Hemoglobin; †DM2: Type 2 diabetes mellitus; ‡P: Percentile; §AUC ROC: area under the ROC curve; ||HR Risk Ratio; ¶IC: Confidence interval; **η2: Cohen correlation; ††OR: Odds Ratio; ‡RR: Relative Risk

**Figure 4:**
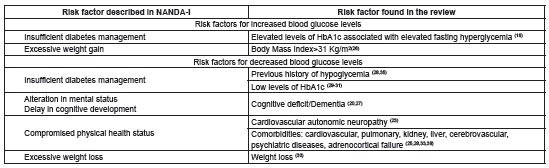
Correspondences between NANDA-I risk factors for risk for unstable blood glucose level in people with type 2 diabetes mellitus, and those identified in a literature review. São Paulo, 2015.

Figure 4 describes the risk factors for unstable blood glucose levels identified in this review, and possible correspondences with six risk factors proposed by NANDA-I: insufficient diabetes management; excessive weight gain, compromised physical health status; alteration in mental status; delay in cognitive development; and excessive weight loss.

**Figure 5:**
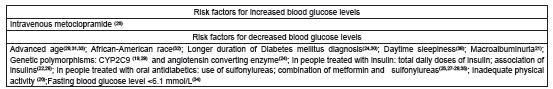
Risk factors for risk for unstable blood glucose level in people with type 2 diabetes mellitus identified in a literature review, without correspondence to NANDA-I risk factors. São Paulo, 2015.

Figure 5 describes additional risk factors for which no correspondences were established with the NANDA-I classification.

## Discussion

This review identified the risk factors for unstable blood glucose levels in people with type 2 DM. Most articles studied the reduction of blood glucose levels, especially severe hypoglycemia. 

Hypoglycemia is the most common acute variation in diabetic patients, especially in type 1 and type 2 diabetes, and type 2 insulin-treated diabetes. It is defined as blood glucose levels lower than 70 mg/dL. Severe hypoglycemia, i.e., hypoglycemia requiring administration of carbohydrates, glucagon, or other resuscitative actions, is a potentially fatal condition^39^.

It was possible to establish a correspondence of the risk factors identified in the review with six NANDA-I risk factors: insufficient diabetes management; excessive weight gain, compromised physical health status; alteration in mental status; delay in cognitive development; and excessive weight loss. Although it was not possible to establish a correspondence with the other NANDA-I risk factors, the authors of this review do not think they should be disregarded. Clinical experience shows that these risk factors can contribute to variations in blood glucose levels in people with type 2 DM.

One exception is the risk factor, Rapid growth period. Type 2 DM is more common after 40 years old, whereas type 1 DM affects mainly children and adolescents^39^. Thus, this risk factor seems more suitable for people with type 1 DM.

A correspondence was found between the NANDA-I risk factor, insufficient diabetes management, with high or low HbA1c and a previous history of hypoglycemia. The management of a chronic disease, such as type 2 DM, extrapolates the biological aspects^40^, however the biological markers are still considered the gold standard for its investigation. In the context of type 2 DM, HbA1c is a method that allows for the assessment of glycemic control in the long term^39^. Therefore, the assessment of HbA1c by nurses is not only valuable for the assessment of the history of the person with type 2 DM, but also for the risk assessment of future glycemic variation. 

Compromised physical health status is a NANDA-I risk factor that can be understood, in this context, as the presence of chronic diseases, such as: coronary heart disease, cardiovascular autonomic neuropathy, heart failure, chronic anemia, kidney damage, depression, mood disorders and adrenocortical insufficiency, which may compromise the physical health of people with type 2 DM^25^ ^,^ ^28^ ^,^ ^33^ ^,^ ^38^. In addition to the number of multiple comorbidities, their type and severity can be important influences on prioritizing care for individuals with diabetes, and on the ability of self-care performance for people with DM and, consequently, glycemic variation^41^.

Another factor that can undermine self-care ability, increase the number of medication errors of people with DM, represent associated comorbidity or frailty, and thereby increase the risk of hypoglycemia is daytime sleepiness^38^. However, sleepiness may have different causes, one of which can be hypoglycemia itself.

The NANDA-I risk factors, "alteration in mental status" and "delay in cognitive development", are related to impaired cognitive status and dementia, identified in this review. They can affect the functional ability and self-care of patients with type 2 DM ^(^ ^20^, an essential requirement to prevent episodes of glycemic variation and the emergence of acute and chronic complications related to the disease^42^. 

Weight loss, found in this review can have a correspondence with the NANDA-I risk factor, "excessive weight loss". Weight loss is common in type 2 DM, probably due to the catabolism that characterizes the disease. Additionally, certain drugs used to treat the disease can cause weight loss^43^. In fact, weight loss may be considered a risk factor for glucose variations, especially hypoglycemia, when the given dosage is not adjusted to weight changes.

Another factor associated with a higher likelihood of increasing blood glucose levels was the intravenous use of metoclopramide, which antagonizes the effects of incretins. This interaction induces glucose-dependent insulin secretion and inhibits glucagon secretion, leading to postprandial hyperglycemia^26^ ^,^ ^44^.

Regarding risk factors that increase the likelihood of lowering blood glucose levels, the following were also identified: age; African-American ethnicity; longer duration of diabetes; insulin therapy; therapy with oral antidiabetic agents; macroalbuminuria; inadequate physical activity; and genetic factors.

Advanced age is associated with an increased risk of hypoglycemia, due to factors such as: adverse effects of medication, poor nutrition, cognitive impairment, renal failure, autonomic dysfunction, and long-term DM^45^. Also regarding age, several studies selected in this review emphasize caution in the use of sulfonylureas in the elderly, because they have greater chances of developing severe hypoglycemia^25^ ^,^ ^27^.

Regarding race, one study showed that the hypoglycemic risk in African-Americans was 2.5 to 3 times that of Caucasians, especially in the first days of hospitalization. The authors of the study explain the finding by the likely lack of adherence to treatment at home and the diminished capacity of the HbA1c test to accurately assess glycemic control in African-Americans, environmental factors and lifestyle^32^. As only one study found a relationship between race and risk of hypoglycemia, this result should be interpreted with caution.

The complexity of treatment regimens with insulin, associated with the need for greater attention to glycemic control, may explain the higher incidence of hypoglycemic events in patients under this type of treatment^22^ ^,^ ^33^. The use of simpler regimens and insulin analogs can minimize these risks^46^.

With regard to oral antidiabetics, the American Diabetes Association and the European Association for the Study of Diabetes reinforce that it is necessary to personalize glucose control, by balancing benefits and risks, taking into account the adverse effects of hypoglycemic agents, age and health status, among other factors. The side effects of these medications may lead to the risk of hypoglycemia, especially when associated with others^47^.

The recommendation to personalize glucose control is reinforced by the fact that genetic factors have also been found to be precipitants for hypoglycemic events. Carriers of the CYP2C9 variant allele may be more likely to experience mild attacks of hypoglycemia during treatment with oral antidiabetic sulfonylureas^19^. Individuals with type 2 DM with these polymorphisms may respond more frequently with hypoglycemia.

Although macroalbuminuria is a recognized marker of glomerular injury, the underlying mechanisms that could explain their relationship with hypoglycemia are not yet fully understood^21^. However, when individuals with type 2 DM have diabetic nephropathy with macroalbuminuria, nursing attention towards the possibility of hypoglycemia should be increased.

Inadequate exercise was found to be a risk factor for severe hypoglycemia. The causes include: reducing food intake; longer intervals between meals and exercise; unexpected increase of exercise intensity or duration; increased insulin absorption (depending on location and time of application); summed effect of hypoglycemic agent or insulin with exercise. There are also other situations in which the risk of hypoglycemia increases, such as alcohol abuse or gastrointestinal disorders, such as diarrhea and vomiting. Since physical activity is an important aspect in the treatment of DM because it promotes a better sensitivity of tissues to insulin, there should be attention to adjustment of antidiabetic medications, greater glycemic control, and an analysis of the need for carbohydrate intake to perform physical activities without hypoglycemic episodes^37^. 

This review is limited by the restriction of the search regarding year of publication, idiom and primary studies, and the non-inclusion of gray literature (publication bias), which may have contributed to a failure to identify other risk factors. Additionally, the heterogeneity of the articles was verified empirically by the authors, based on the different methodological characteristics, and did not allow for data integration or conducting a meta-analysis.

## Conclusion

This review updated the existing knowledge on risk factors for the diagnosis, *risk for unstable blood glucose level* . Nineteen risk factors for *risk for unstable blood glucose level* in patients with type 2 DM were identified. Of those, 11 were not included in the NANDA-I diagnostic classification. It is believed that the ND concept analysis associated with the extension of the present review, as well as the development of conceptual and operational definitions, may contribute to the studies of this phenomenon.

The identified risk factors can help nurses in clinical practice to plan and implement care strategies to improve the health outcomes of individuals with type 2 DM at risk of hypo- or hyper-glycemia. Nurse educators can use up-to-date content regarding risk factors for teaching undergraduate students regarding care for individuals with type 2 DM.
